# Compound heterozygous *CASQ2* mutations and long‐term course of catecholaminergic polymorphic ventricular tachycardia

**DOI:** 10.1002/mgg3.323

**Published:** 2017-08-22

**Authors:** Katherine Josephs, Kunjan Patel, Christopher M. Janson, Cristina Montagna, Thomas V. McDonald

**Affiliations:** ^1^ Department of Genetics Albert Einstein College of Medicine Bronx New York; ^2^ Department of Pediatrics (Cardiology) Albert Einstein College of Medicine & Montefiore Medical Center Bronx New York; ^3^ Department of Medicine (Cardiology) Albert Einstein College of Medicine & Montefiore Medical Center Bronx New York; ^4^ Department of Molecular Pharmacology Albert Einstein College of Medicine Bronx New York

**Keywords:** Atrial fibrillation, autosomal recessive, calsequestrin‐2, catecholaminergic polymorphic ventricular tachycardia, alternative splicing

## Abstract

**Background:**

Catecholaminergic polymorphic ventricular tachycardia (CPVT) is a potentially lethal inherited cardiac disorder characterized by episodic ventricular tachycardia during adrenergic stimulation. It is associated with significant morbidity and mortality. Knowledge of the underlying genetic cause, pathogenesis, and the natural history of the disease remains incomplete. Approximately 50% of CPVT cases are caused by dominant mutations in the cardiac ryanodine receptor (*RYR2*) gene, <5% of cases are accounted for by recessive mutations in cardiac calsequestrin (*CASQ2*) or Triadin (*TRDN*).

**Methods:**

We report a family with two *CASQ2* gene mutations. A research‐based next‐generation sequencing (NGS) initiative was used in a patient with a severe CPVT phenotype and her clinically unaffected son. Reverse transcription polymerase chain reaction (RT‐PCR) from platelet RNA was used to assess the consequences of predicted splice variants.

**Results:**

NGS revealed that the proband carried a novel c.199C>T (p.Gln67*) mutation and a previously reported splice site mutation c.532+1G>A in *CASQ2*. Her son is a heterozygous carrier of the c.199C>T (p.Gln67*) mutation alone and the proband was compound heterozygous at *CASQ2*. RNA analysis demonstrated that the splice site mutation results in the retention of intron 3 with no full‐length *CASQ2* mRNA.

**Conclusion:**

This study describes a novel CPVT genotype and further characterizes the effect of a previously reported *CASQ2* splice site mutation. The long‐term follow‐up of 23 years since first symptom provides additional insight into the natural history of *CASQ2*‐associated CPVT.

## Introduction

Catecholaminergic polymorphic ventricular tachycardia (CPVT) is a rare, inherited cardiac disease that predisposes to malignant arrhythmias and sudden death (Behere and Weindling 2016). It is characterized by episodic polymorphic or bidirectional ventricular tachycardia in individuals with otherwise structurally normal hearts (Behere and Weindling 2016). Arrhythmias usually occur under conditions of beta‐adrenergic stimulation, such as exercise or stress (Leenhardt et al. 1995). Although rare, it can be devastating, with early reports estimating mortality rates of 30% by 20–30 years of age (Swan et al. 1999).

Approximately 50% of CPVT cases stem from autosomal dominant, gain‐of‐function mutations in the cardiac ryanodine receptor (*RYR2,* OMIM accession 180902) gene (Behere and Weindling 2016). Recessively inherited cardiac calsequestrin (*CASQ2*, OMIM accession 114251) mutations account for much fewer cases. These are predominately homozygous loss‐of‐function mutations described in consanguineous families (Lahat et al. 2001). Only a few compound heterozygous mutations and one dominant mutation have been found in the *CASQ2* gene (De la Fuente et al. 2008; Gray et al. 2016).

Despite significant progress uncovering the genetic basis of *CASQ2*‐associated CPVT, less is known about its natural history. Treatment is largely based on evidence from CPVT patients with *RYR2* mutations (De la Fuente et al. 2008). The family in the present report harbors two *CASQ2* mutations identified through research‐based next‐generation sequencing (NGS). The proband is compound heterozygous for both a novel stop mutation and a previously reported splice site mutation. We describe the severe phenotype and long‐term clinical course associated with this novel genotype. In addition, we have employed reverse transcription polymerase chain reaction (RT‐PCR) on RNA extracted from platelets to investigate the effect of this splice site mutation on the resulting mRNA.

## Material and Methods

### Clinical evaluation

Clinical evaluation of the proband and her child included detailed personal and family history, physical examination, 12 lead electrocardiogram (ECG), echocardiogram, exercise stress test, and Holter monitoring. The study was approved by the Albert Einstein College of Medicine Committee on Clinical Investigations (IRB 2010‐548).

### DNA collection and analysis

The proband's DNA was collected, sequenced, and analyzed as part of a larger study developing a targeted multi‐disorder high‐throughput sequencing assay. The methods have been detailed previously (Delio et al. 2015). In brief, ~10 mL of whole blood was collected from the patient and genomic DNA was purified using the Puregene Genomic DNA Purification kit (Gentra, Minneapolis, MN, USA). Next, all coding, untranslated regions (UTR) and flanking intronic regions of 650 known disease‐associated genes were targeted. Within this panel there were 154 cardiac disease‐associated genes (listed in Table S1). Targeted capture‐sequencing was done using the Roche‐NimbleGen EZ SeqCapV3 capture system and sequenced on the Illumina Hiseq 2500 Rapid Run platform. Sequence reads were analyzed using a custom in‐house generated analytical pipeline (Delio et al. 2015). We sequenced eight controls and one HapMap sample to assess the precision of the panel in identifying known variants. Samples were analyzed for copy number variation using the Affymetrix Genome Wide SNP Array 6.0. Sanger sequencing was used to confirm mutations. All mutations discovered were subsequently validated in a CLIA‐approved commercial laboratory.

### Platelet RNA extraction and cDNA synthesis

The patient's platelets were separated from 3 mL whole blood using OptiPrep^TM^ reagent. Platelet mRNA was extracted using the RNeasy Mini Kit (Qiagen, Germantown, MD, USA). cDNA was synthesized from 1ug of total RNA using random hexamers and SuperScriptIII (Invitrogen, Walthan, MA, USA).

### *CASQ2*‐specific cDNA and RT‐PCR sequencing

Gene‐specific cDNA was synthesized using *CASQ2* Exon 9 reverse primer (CTCAGATCGGGGTTGTCAGT) and SuperScript III (Invitrogen). Primers flanking the splice site mutation (c.532+1G>A) were then used for amplification using RT‐PCR. The primers were exon 3 forward (GAGTTTGATGGCGAGTTTGC) and exon 6 reverse (TCCACCAGCTCCTCTTCTGT). The resulting PCR product fragments were gel purified using the QIAquick Gel Extraction Kit (Qiagen) and subjected to Sanger sequencing on the ABI 3730 sequencer.

## Results

### Clinical presentation

**Figure 1:**
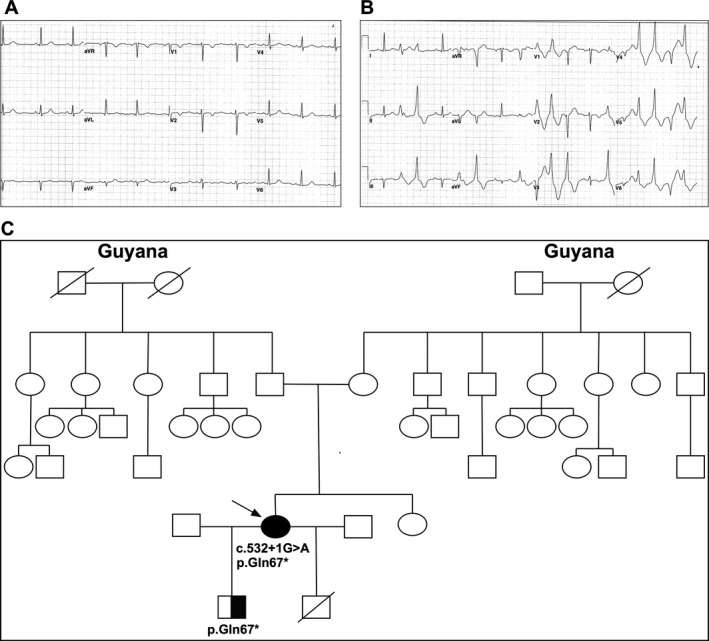
(A) Proband's resting ECG shows normal sinus rhythm. (B) Proband's ECG during an exercise stress test shows consecutive premature ventricular complexes with a bidirectional pattern and nonsustained runs of polymorphic ventricular tachycardia. (C) Family pedigree. Squares indicate males; circles, females; line through a symbol, deceased individual; arrow, patient; solid black symbol, presence of both *CASQ2* mutations; half black symbol, presence of one *CASQ2* mutation; open symbols, clinically unaffected and untested individuals.

The proband presented at age 11 with a history of palpitations and syncope since the age of 3. She recalled several fainting episodes while playing field hockey. Syncopal episodes always occurred with exercise with no symptoms at rest. ECG during exercise revealed multiform and consecutive premature ventricular complexes (Fig. 1A,B). She was provisionally diagnosed with either long QT syndrome (due to intermittent QT interval prolongation seen on some ECGs) or CPVT and was subsequently treated with beta‐adrenergic receptor blocker therapy.

### Family history

The proband has no known family history of syncope, seizures, sudden, or unexplained death. Both of her parents are from Guyana of Indian ancestry and are alive and well without cardiovascular symptoms in their fifth decade. There is no history of consanguinity (Fig. 1C). At age 24, she had a premature delivery (25 weeks gestation) by caesarian section for fetal distress; the baby did not survive. Three years later, she gave birth to a full‐term healthy boy by caesarian section. There were no problems reported during this pregnancy or delivery. Extensive family history failed to reveal any episodes of sudden death, seizures, syncope, or early unexplained death. Older generations lived into sixth or seventh decades with causes of death reported due to diabetes or cancer. No genetic sequencing was available from extended family members.

### Cardiac arrhythmia gene analysis

By NGS analysis, the proband was found to be compound heterozygous for mutations in the *CASQ2* gene (Locus Reference Genomic sequence LRG_404; NCBI Reference Sequence: NM_001232.3) (Fig. S1). We identified one novel mutation, c.199C>T (p.Gln67*), in exon 1 which results in the substitution of glutamine and a stop codon interrupting the reading frame. We also identified a splice site mutation, c.532+1G>A. This mutation involves an intronic base‐pair change (from G to A) 1 bp downstream from exon 4. This same nucleotide change has been previously reported in a consanguineous CPVT family. Although the underlying mechanism was unknown at the time, it was predicted to alter normal splicing (Postma et al. 2002). No mutations were detected in *RYR2* or in other genes linked to inherited arrhythmia syndromes. An additional variant was observed in the proband in the *MYH7* gene (NM_000257.3(MYH7):c.4472C>G (p.Ser1491Cys), dbSNP number rs3729823) in the heterozygous state. This variant has been classified as benign in ClinVar by expert review panel as of 6 December 2016 (Variation ID: 43020). Using cascade testing we established that the proband's unaffected son is a heterozygous carrier of the c.199C>T (p.Gln67*) mutation alone. This confirms that both of the proband's *CASQ2* alleles each carry a separate mutation.

### Platelet RT‐PCR

**Figure 2:**
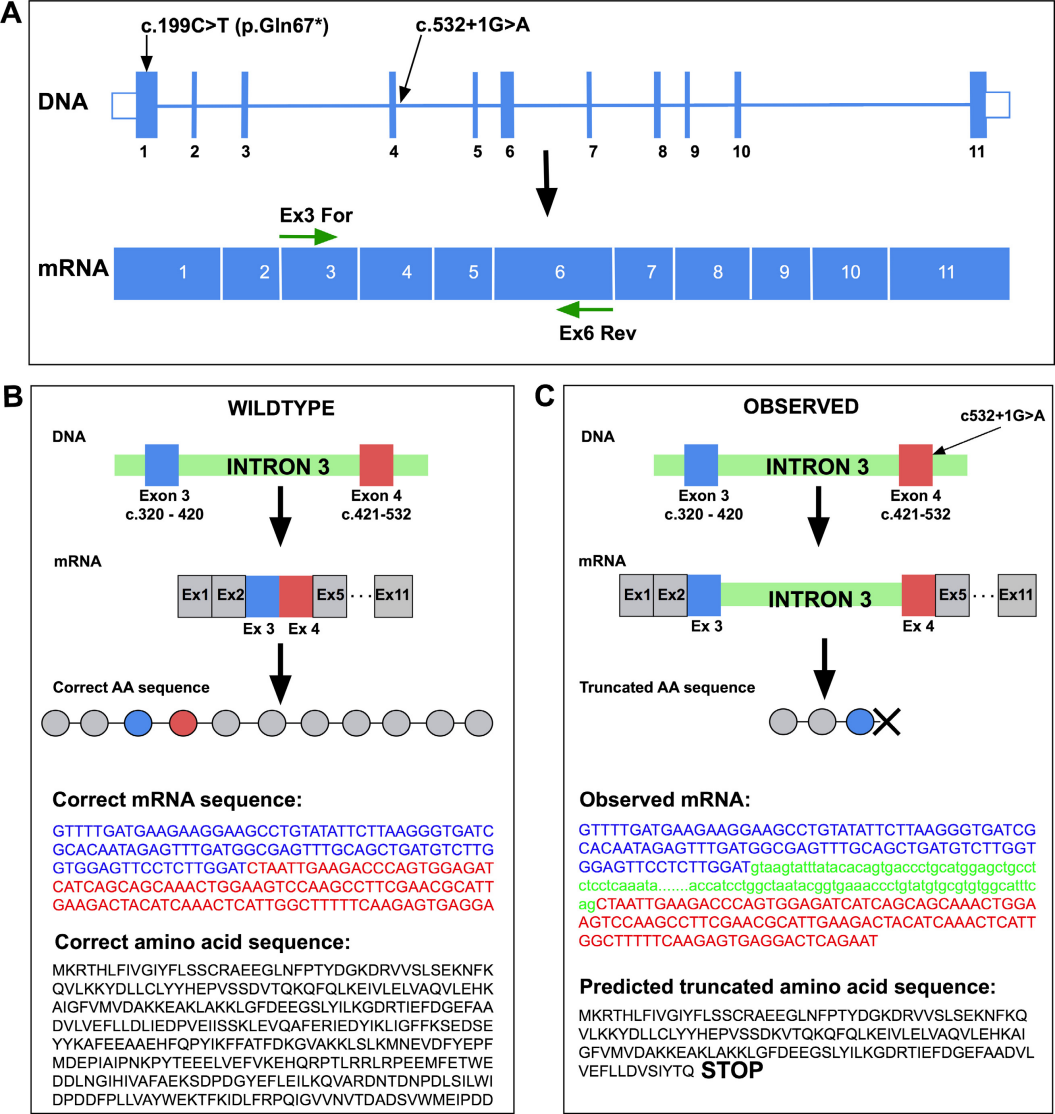
(A) Model of *CASQ2* DNA and mRNA transcript. Solid blue boxes represent exons, numbered 1–11. DNA transcript: relative position of mutations is shown, P.Gln67* in exon 1, c.532+1G>A, an intronic mutation occurring 1 bp after exon 4. mRNA transcript: green arrows indicate primers (exon 3 forward, exon 6 reverse). (B) Cartoon depicting the effect of splice site mutation c532+1G>A. Left panel: wild‐type *CASQ2*. Intron 3 (green) is spliced out during transcription. Right panel: the observed effect of the mutation. Intron 3 is retained resulting in a truncated amino acid sequence.

To further investigate the effect of the splice site mutation (c.532+1G>A), we first determined if it was possible to detect expression of *CASQ2* within platelet mRNA. CASQ1 and low‐level *CASQ2* expression have been previously observed in human and rat platelets (Zhu et al. 2012). After generating cDNA from the patient's platelet RNA, *CASQ2* cDNA was amplified using specific primers flanking the splice site mutation (Fig. 2A). Sequencing revealed a retained intron 3 and no wild‐type full‐length *CASQ2* mRNA (Fig. 2B,C). We hypothesize that the mRNA produced from both the proband's *CASQ2* alleles will be targeted for nonsense mediated decay (NMD) resulting in little or no functional calsequestrin protein in the heart (Lykke‐Andersen and Jensen 2015).

### Long‐term clinical course and follow‐up

On the initial presentation to our institution at age 13 years, the proband had a QTc interval that was prolonged after her treadmill stress test. Accordingly, she was initially diagnosed variably with long QT syndrome or CPVT. She was treated with metoprolol but breakthrough symptoms were managed by the sequential addition of mexiletine and propafenone. At the age of 14, she had an implantable cardioverter‐defibrillator (ICD) inserted due to documented episodes of polymorphic ventricular tachycardia. She continued to experience symptoms and received numerous appropriate ICD shocks. Her drug regimen required regular adjustments, and now includes metoprolol (100 mg QD), mexiletine (250 mg TID), and propafenone (150 mg TID). On this regimen, she remained free of symptoms and ICD shocks for several years. However, at age 31, during a time of unusual emotional stress, she experienced palpitations without syncope that was followed by five successive ICD shocks. ICD interrogation revealed an episode of atrial fibrillation with rapid ventricular response.

Although her medication was not altered, the patient admits she self‐administers additional metoprolol when she feels stress. No further arrhythmic episodes have occurred.

Over the past 5 years, her ECGs have shown development of low QRS voltage in the precordial leads, increased QRS duration, and right bundle branch block (Fig. S2A). These changes could be pharmacologically driven. Such ECG changes have not been described to date for CPVT. Given the scarcity of homozygous CASQ2 CPVT patients with long‐term follow‐up, it is unclear if other cardiac manifestations may ensue. Calsequestrin is a low‐affinity, high‐capacity calcium binder that polymerizes in the cardiac sarcoplasmic reticulum. It is unknown if complete loss of CASQ2 eventually has other effects on cardiomyocytes and cardiac function. Her most recent echocardiogram showed normal systolic and diastolic function, with moderate tricuspid valve regurgitation (Fig. S2B–E).

The proband's child (who harbors the c.199C>T (p.Gln67*) mutation), is now 6 years old and has been asymptomatic. His ECG is normal at rest (Fig. S3) and an echocardiogram showed normal systolic and diastolic function. Holter monitoring was also normal and revealed a peak heart rate of 173 bpm with no ventricular ectopy.

## Discussion

This study has identified a compound heterozygous *CASQ2* mutation in a patient with CPVT and no family history. She carries a previously reported splice site mutation (c.532+1G>A) on one allele and a novel stop mutation (c.199C>T (p.Gln67*)) on the other. The patient's clinical course has been severe: her symptoms began at a very young age, she requires triple medical therapy and she has had an ICD in situ for 19 years. The stop mutation alone has been identified in her son, who is now 6 years old and remains asymptomatic with no evidence of ventricular arrhythmia. CPVT in this family appears to be inherited recessively as predicted for *CASQ2* mutations with loss‐of‐function.

Autosomal recessive (either homozygous or compound heterozygous), loss‐of‐function *CASQ2* mutations have been associated with a severe CPVT phenotype. Heterozygous carriers of these mutations can remain asymptomatic or have a much milder clinical course. This has been reproduced in mouse models (Chopra et al. 2007).

To date, there has only been limited success in defining the mechanism underlying CPVT caused by recessive mutations in *CASQ2*. *CASQ2* works alongside *RYR2* with the proteins junctin (gene ASPH, OMIM accession 600582) and triadin (gene *TRDN*, OMIM accession 603283) to form the calcium release unit (CRU) (Bal et al. 2010) (Faggioni et al. 2012) (Fig. S2F). The CRU provides precise control of Ca^2+^ release during excitation–contraction coupling in the cardiac cycle. To achieve this, Ca^2+^ must be buffered to allow its measured release and reuptake in the SR. *CASQ2* is the principle Ca^2+^ buffer and has a role in modulating *RYR2* activity (Bal et al. 2010; Faggioni et al. 2012).

Experimental studies indicate that upon Ca^2+^ binding, CASQ2 proteins dimerize and then polymerize in a linear fashion (Bal et al. 2010). Disrupted *CASQ2* polymerization dynamics may impair Ca^2+^ handling during increased physiological demands which could explain the CPVT phenotype (Bal et al. 2010). This has been further supported by the study of a novel dominant *CASQ2* mutation. Predictive modeling of this mutation has suggested it might disrupt *CASQ2* assembly (Gray et al. 2016).

Both disruption of polymerization dynamics and decreased protein levels may contribute to the CPVT phenotype but could they also impact myocyte stability and structure? Young mice with *CASQ2* mutations have normal cardiac morphology; however, by 35 weeks these mice can exhibit both cardiac hypertrophy and left ventricular dysfunction (Song et al. 2007). Whether *CASQ2* mutations can promote similar effects in humans is uncertain given limited data on the long‐term outcomes in these patients. Our patient's ECGs have begun to show low QRS voltage and intraventricular conduction delay. Currently, she has no new cardiac symptoms and a repeat echocardiogram was unchanged but she will continue to be monitored for developing cardiomyopathy.

Atrial fibrillation has been described in CPVT patients and it has been shown to be more easily inducible in *CASQ2* knockout mice than in wild‐type mice (Faggioni et al. 2014). The inducibility can be diminished by propafenone, a dual sodium channel and RYR inhibitor (Faggioni et al. 2014). The proband in this report exhibited spontaneous atrial fibrillation in adulthood briefly, during stress but has otherwise been absent on a medical regimen that includes propafenone.

The splice site substitution found here was first described as a homozygous mutation in a consanguineous family (Postma et al. 2002). In 2002, the authors were unable to confirm any alteration in *CASQ2* mRNA splicing. Now, through amplification of *CASQ2* mRNA from our patient's platelets, we have shown this mutation causes retention of intron 3 with production of no measurable wild type, full‐length mRNA. The nonsense transcript is likely recognized and degraded via NMD. Although we need myocytes to definitively conclude the level of *CASQ2* protein in the patient's heart, our results certainly suggest that this splice site mutation, coupled with the stop mutation, will be enough to render the patient with no *CASQ2* protein.

*CASQ2*‐induced CPVT is rare and there are little data available on the long‐term clinical outcomes. More information is needed to improve treatment, surveillance, and genetic counseling for these patients. This family has been followed up for 23 years since her first symptom providing key insights into the disease's natural history. Using our research‐based target panel, we confirmed the CPVT diagnosis and identified a novel *CASQ2* stop mutation. We have shown that it is possible to amplify *CASQ2* mRNA from platelets allowing new insights into splice site mutation effects. Finally, because of this work, additional at‐risk family members have now been offered cascade testing.

## Conflict of Interest

The authors have no conflicts of interest.

## Supporting information


**Figure S1.** Integrative Genomics Viewer snapshot showing the two heterozygous *CASQ2* mutations in this patient. Both mutations were confirmed using Sanger sequencing.

**Figure S2.** (A) Recent resting ECG from the proband showing low QRS voltage. (B–E) Echocardiogram images from the proband. (B) Parasternal short‐axis view, systole. (C) Parasternal short‐axis view, diastole. (D) Apical four‐chamber view, systole. (E) Apical four‐chamber view, diastole. (F) Model of *CASQ2*. Under resting conditions, there is abundant Ca^2+^ in the sarcoplasmic reticulum. *CASQ2* forms linear polymers and buffers Ca^2+^. Ca^2+^‐induced Ca^2+^ release occurs through *RYR2* channels with reuptake of Ca^2+^ occurring via the SERCA2/PLN complex.

**Figure S3.** An ECG from the proband's son at rest showing normal sinus rhythm.

**Table S1.** Gene Panel used for sequencing of proband.
