# Supplementary figures and images for: Compound heterozygous CASQ2 mutations and long‐term course of catecholaminergic polymorphic ventricular tachycardia
Source: Mol Genet Genomic Med. 2017 Aug 22;5(6):788–94. doi: 10.1002/mgg3.323 (PMC5702571; doi:10.1002/mgg3.323)

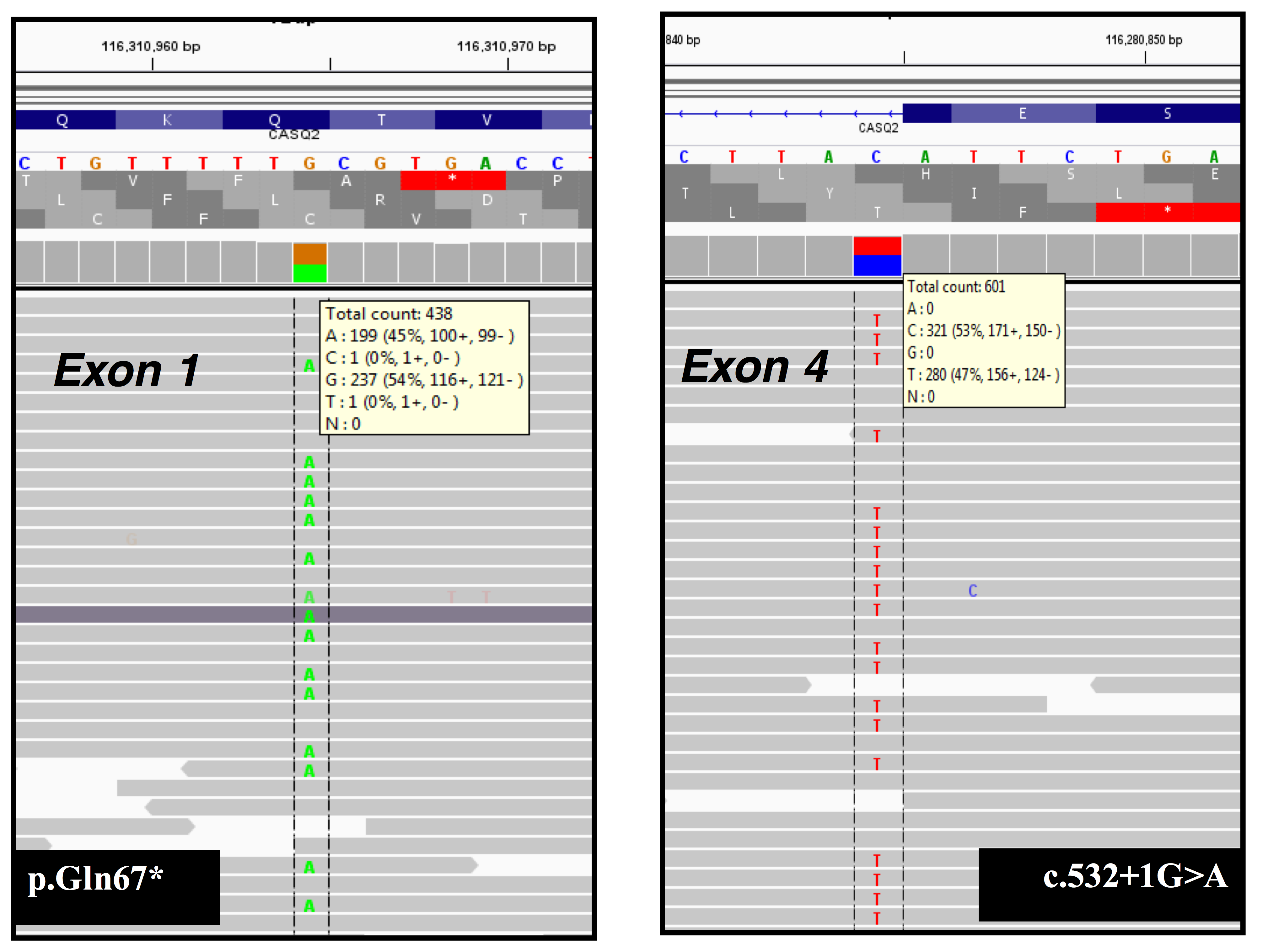

Supplement: Supplementary file 1 — Figure S1. Integrative Genomics Viewer snapshot showing the two heterozygous CASQ2 mutations in this patient. Both mutations were confirmed using Sanger sequencing. [file MGG3-5-788-s001.tiff]

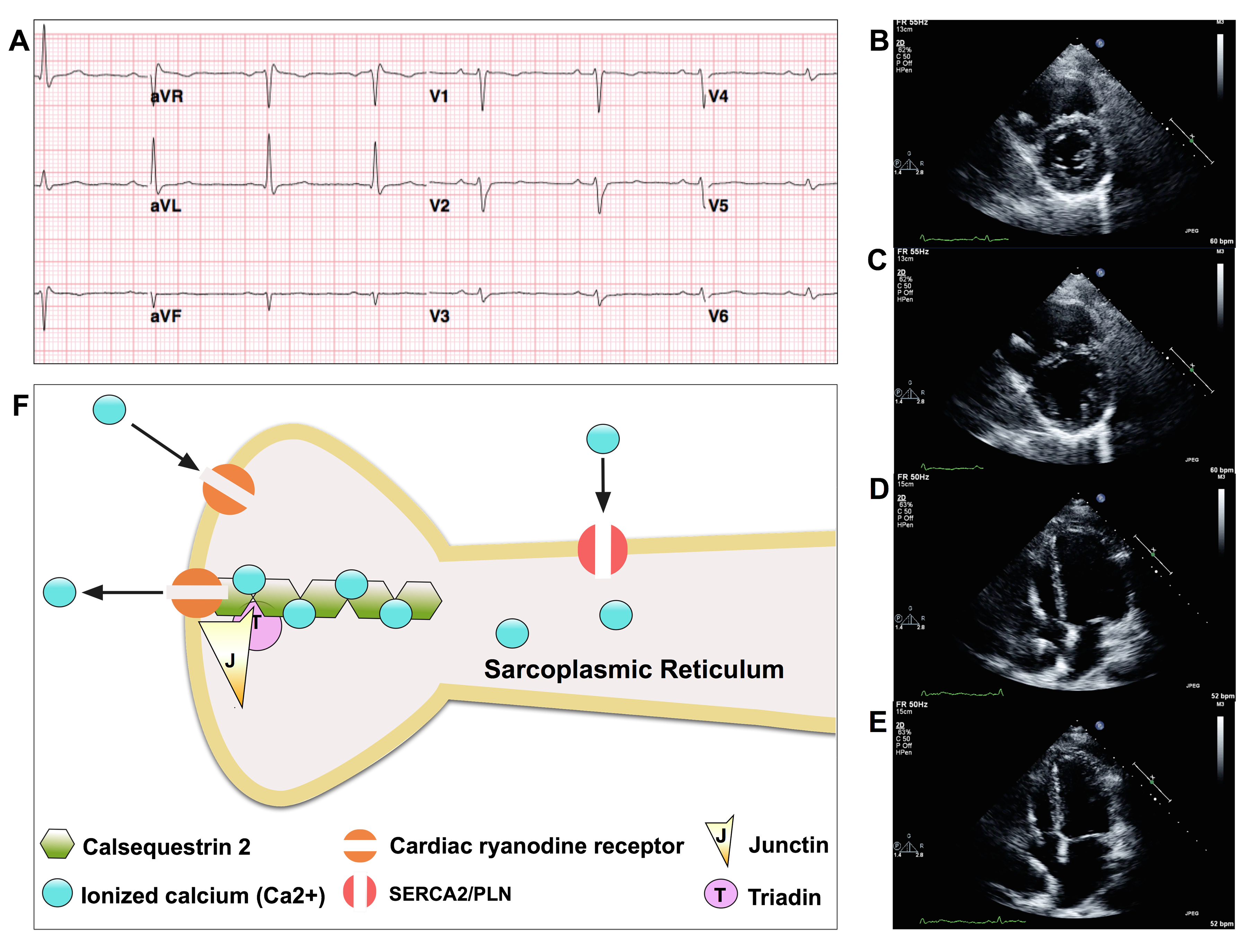

Supplement: Supplementary file 2 — Figure S2. (A) Recent resting ECG from the proband showing low QRS voltage. (B–E) Echocardiogram images from the proband. (B) Parasternal short‐axis view, systole. (C) Parasternal short‐axis view, diastole. (D) Apical four‐chamber view, systole. (E) Apical four‐chamber view, diastole. (F) Model of CASQ2. Under resting conditions, there is abundant Ca2+ in the sarcoplasmic reticulum. CASQ2 forms linear polymers and buffers Ca2+. Ca2+‐induced Ca2+ release occurs through RYR2 channels with reuptake of Ca2+ occurring via the SERCA2/PLN complex. [file MGG3-5-788-s002.tiff]

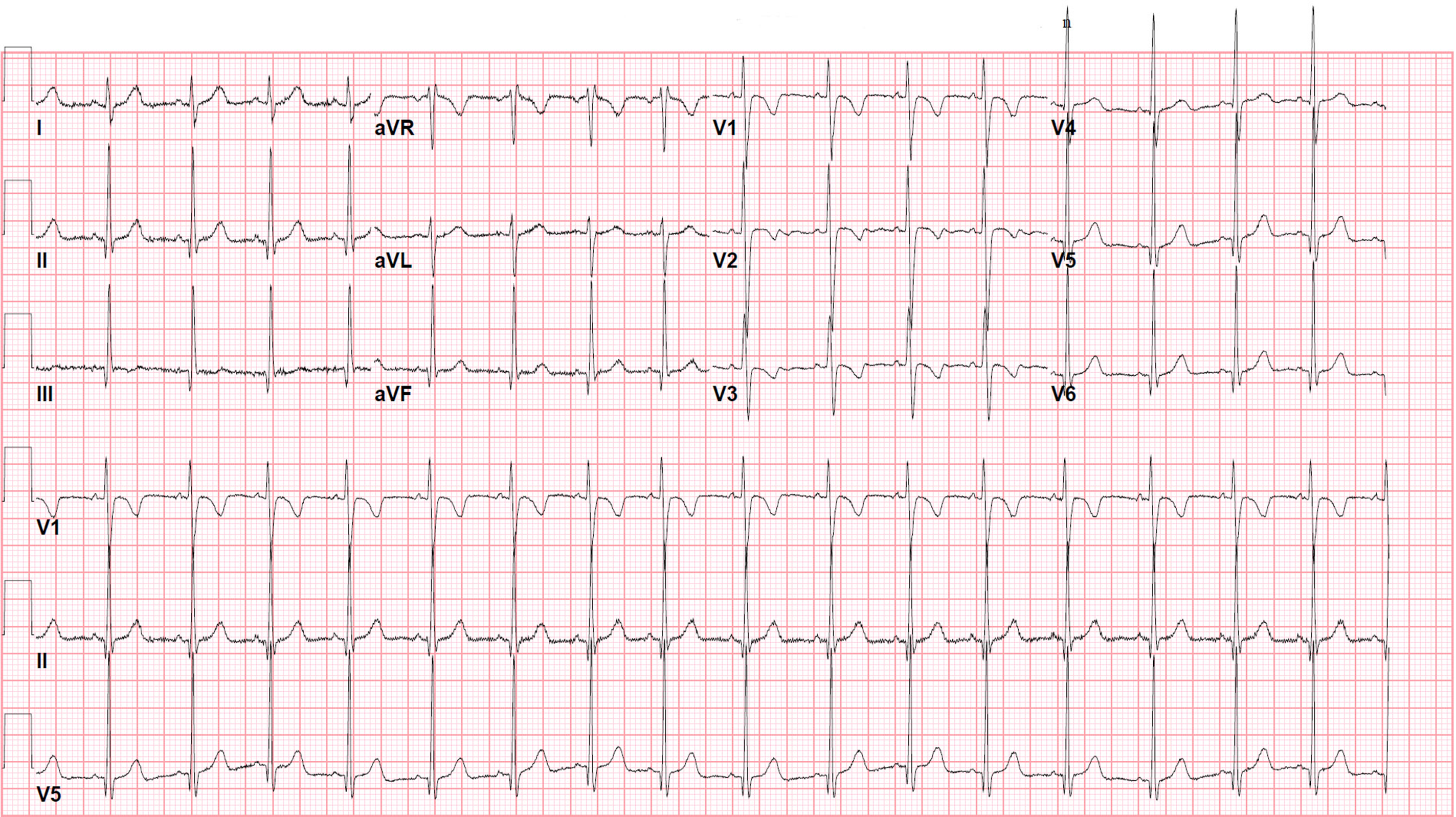

Supplement: Supplementary file 3 — Figure S3. An ECG from the proband's son at rest showing normal sinus rhythm. [file MGG3-5-788-s003.tiff]
